# Children’s syntax is supported by the maturation of BA44 at 4 years, but of the posterior STS at 3 years of age

**DOI:** 10.1093/cercor/bhac430

**Published:** 2022-11-20

**Authors:** Cheslie C Klein, Philipp Berger, Tomás Goucha, Angela D Friederici, Charlotte Grosse Wiesmann

**Affiliations:** Department of Neuropsychology, Max Planck Institute for Human Cognitive and Brain Sciences, Stephanstraße 1a, Leipzig 04103, Germany; Research Group Milestones of Early Cognitive Development, Max Planck Institute for Human Cognitive and Brain Sciences, Stephanstraße 1a, Leipzig 04103, Germany; Department of Neuropsychology, Max Planck Institute for Human Cognitive and Brain Sciences, Stephanstraße 1a, Leipzig 04103, Germany; Research Group Milestones of Early Cognitive Development, Max Planck Institute for Human Cognitive and Brain Sciences, Stephanstraße 1a, Leipzig 04103, Germany; Department of Neuropsychology, Max Planck Institute for Human Cognitive and Brain Sciences, Stephanstraße 1a, Leipzig 04103, Germany; Department of Neuropsychology, Max Planck Institute for Human Cognitive and Brain Sciences, Stephanstraße 1a, Leipzig 04103, Germany; Research Group Milestones of Early Cognitive Development, Max Planck Institute for Human Cognitive and Brain Sciences, Stephanstraße 1a, Leipzig 04103, Germany

**Keywords:** Broca’s area, cortical maturation, early childhood, language development, syntax development

## Abstract

Within the first years of life, children learn major aspects of their native language. However, the ability to process complex sentence structures, a core faculty in human language called syntax, emerges only slowly. A milestone in syntax acquisition is reached around the age of 4 years, when children learn a variety of syntactic concepts. Here, we ask which maturational changes in the child’s brain underlie the emergence of syntactically complex sentence processing around this critical age. We relate markers of cortical brain maturation to 3- and 4-year-olds’ sentence processing in contrast to other language abilities. Our results show that distinct cortical brain areas support sentence processing in the two age groups. Sentence production abilities at 3 years were associated with increased surface area in the most posterior part of the left superior temporal sulcus, whereas 4-year-olds showed an association with cortical thickness in the left posterior part of Broca’s area, i.e. BA44. The present findings suggest that sentence processing abilities rely on the maturation of distinct cortical regions in 3- compared to 4-year-olds. The observed shift to more mature regions involved in processing syntactically complex sentences may underlie behavioral milestones in syntax acquisition at around 4 years.

## Introduction

Language comprehension and production are among the most demanding cognitive abilities to be acquired during development. In order to gain full proficiency in their language, children have to successfully master each of its modular components and learn to integrate them altogether. Already within the first year of life, children learn to segment the incoming speech stream to phonological word forms and to associate them with meaning stored in the mental lexicon (Speer and Ito 2009; Bergelson and Swingley 2012). Before the age of two, children can use prosodic cues for the detection of phrase boundaries and can organize words into morphosyntactic categories (Soderstrom 2003; Höhle et al. 2004). However, the ability to engage in the rule-based formation of words into a hierarchical sentence structure, referred to as syntax, develops comparably slowly and is not fully mastered until young adulthood at around the age of 20 years (Nippold et al. 2005; Skeide and Friederici 2016; Friederici 2020). Between 3 and 4 years of age, developmental milestones are observed in the ability to process and produce complex sentence structures (Fox and Grodzinsky 1998; Akhtar 1999; Tomasello and Brooks 1999; Guasti 2002; Kauschke 2012). Particularly, while at 3 years, children reliably process the canonical word order of their language (Akhtar 1999; Dittmar et al. 2008; Schipke et al. 2012), more complex syntactic phrases, such as long-distance wh-questions, passive constructions, and subordinate clauses, are not fully acquired before the fourth birthday (Fox and Grodzinsky 1998; Tomasello and Brooks 1999; Guasti 2002; Kauschke 2012). What are the neural maturational processes that underlie this critical step in human language development? In the present study, we set out to investigate the maturation of brain structure in the critical age range between 3 and 4 years that support the acquisition of syntactically complex sentences.

In adults, language processing has been found to demand multiple brain areas to interact, together forming what has been referred to as the language network in the human brain (Friederici 2002). The core of this network includes Broca’s area, which consists of the pars opercularis (BA44) and the pars triangularis (BA45) in the left inferior frontal gyrus (IFG; Skeide and Friederici 2016), and Wernicke’s area in the superior temporal lobe (DeWitt and Rauschecker 2013). In the mature brain, BA45 in the anterior part of the IFG is functionally involved in the processing of lexical meaning (semantics) together with the temporal lobe. Conversely, BA44 in the posterior part of the IFG is involved in the integration of syntactically complex sentences in a multitude of studies (Friederici et al. 2000, 2006; Friederici 2002, 2011; Makuuchi et al. 2009; Newman et al. 2010; Goucha and Friederici 2015; Zaccarella et al. 2017). For example, Goucha and Friederici (2015) reported an activation in both parts of the left IFG when adult participants heard sentences consisting of words without meaning but with correct syntactic order and derivational and inflectional morphological elements. After removing the meaning-conveying derivational morphological elements leaving only elements that convey structural cues, the authors found an effect solely in BA44 suggesting that this area is crucial for syntactic processing.

Although adults show dissociable functional representations of syntactic and semantic features in the brain (Skeide and Friederici 2016), behavioral and imaging studies in children indicate that this functional segregation is not completed until late childhood (Friederici 1983; Brauer and Friederici 2007; Nuñez et al. 2011; Knoll et al. 2012; Skeide et al. 2014; Wu et al. 2016; Xiao et al. 2016; Strotseva-Feinschmidt et al. 2019). Children’s syntactic abilities show a strong correlation with their semantic knowledge, which might also predict grammatical proficiency during the first three years of language development (Marchman and Bates 1994; Moyle et al. 2007; Snedeker et al. 2007, 2012). This has led to the hypothesis that grammar and lexicon are not dissociable during processing and exhibit a bidirectional relationship in language acquisition (Marchman and Bates 1994; Bates and Goodman 1997; Frank et al. 2021). In line with this suggestion, it was found that children at least until the age of 7 years strongly rely on word meaning to successfully process complex sentences (Dittmar et al. 2008; Skeide et al. 2014; Skeide and Friederici 2016). For example, while 3-year-old children have difficulties processing object-initial sentences when both the agent and patient are animate (Schipke et al. 2012), they perform above chance when only the agent of the sentence is animate, providing them with a semantic cue to resolve the verb argument structure (Strotseva-Feinschmidt et al. 2019). Skeide et al. (2014) found that children’s reliance on semantics when processing syntactically complex sentences was reflected in reduced functional segregation in the language network: Children aged 3.9 to 4.11 years did not show separate effects of syntactic complexity or semantic plausibility in their neural activation. Instead, they exhibited an interaction of syntax and semantics in the posterior part of the left superior temporal lobe. The neural activation for processing syntactically complex sentences in left BA44 increased between 4 and 9 years of age (Skeide et al. 2016). An increase in activation in the left IFG with increasing syntactic proficiency was confirmed by a number of functional brain imaging studies from 5 years of age (Brauer and Friederici 2007; Nuñez et al. 2011; Knoll et al. 2012; Wu et al. 2016; Xiao et al. 2016). These studies also underline that the interference of syntax and semantics is reflected in an immature brain activation pattern. In sum, on a brain functional level, these findings show an interaction between syntactic and semantic processes in young children with an increasing spatial segregation during middle childhood. Against the background of a major behavioral improvement in complex sentence comprehension and production, a central question is to what extent the maturation of the left IFG contributes to the change in sentence processing abilities observed between 3 and 4 years. In the present study, we therefore set out to investigate which brain structural changes underlie and precede the increasing functional involvement of the left IFG in sentence processing observed after 4 years of age.

It is known that during brain maturation in early childhood, the human cerebral cortex undergoes complex cytoarchitectonic changes, caused by the proliferation of dendrites, synaptic pruning and myelination (Walhovd et al. 2016). Using structural magnetic resonance imaging (sMRI) as an in vivo reconstruction method, cortical thickness and surface area have been suggested as indices of cortical maturation (Fischl and Dale 2000), providing spatial information about the developmental status of brain regions and networks. Cortical surface area has been shown to expand dramatically across the entire cortex between infancy and adulthood with a peak in late childhood (Li et al. 2014; Lyall et al. 2015). On a regional level, this increase in cortical surface area is positively related to cognitive development in childhood (Walhovd et al. 2016; Cafiero et al. 2019; Grosse Wiesmann et al. 2020). The developmental trajectory of cortical thickness, in contrast, is less clear, as its peak depends on complementary microphysiological processes and is subject to considerable local and interindividual variation (Shaw et al. 2006; Cafiero et al. 2019; Natu et al. 2019; Grosse Wiesmann et al. 2020). A range of studies suggest that cortical thickness peaks around late childhood and thins thereafter, with association cortices showing a later peak than sensory and motor cortices (Sowell 2004; Shaw et al. 2008). Accordingly, depending on the age, cognitive domain, and brain region, cortical thickness has been found to be either positively or negatively related to cognitive function (Shaw et al. 2006; Cafiero et al. 2019; Grosse Wiesmann et al. 2020).

In early preschool age, the relation of cortical brain structure and cognitive function has rarely been studied to date and research on the relation to early language and syntactic development is lacking. In children aged 4 years and older, some evidence exists that language development is related to cortical maturation in the language network, and particularly the left IFG (Sowell 2004; Lu et al. 2006; Richardson et al. 2010; Fengler et al. 2015; Qi et al. 2019, 2021). In addition to a relation of children’s sentence comprehension with cortical structure in the left IFG, Fengler et al. (2015) also found an association in the inferior parietal lobe and posterior superior temporal gyrus in 5- to 8-year-olds. While several of these studies have investigated brain structural changes involved in sentence comprehension from 4 or 5 years (Fengler et al. 2015; Qi et al. 2019, 2021), the maturational changes of those brain structures that support early sentence processing before children start to master more complex sentence structures remain largely unknown. This leaves a gap in the literature on the brain maturational changes underlying the important syntactic milestones achieved between 3 and 4 years of age.

In the present study, we therefore investigate the role of cortical brain maturation in early sentence processing in young 3- and 4-year-old children. We focused on children’s sentence production and comprehension and compared this to their global language abilities. In a preregistered procedure, we extracted these measures from a standardized test of children’s general language development and related them to gray matter maturation indices in regions of the left-lateralized language network. We hypothesized that language performance, in general, would be associated with maturation indices in the entire left language-related brain network (Friederici and Gierhan 2013; Fedorenko and Thompson-Schill 2014). Children’s sentence processing abilities, in contrast, were expected to be specifically related to the maturation of left BA44 with increasing relevance of this region between 3 and 4 years of age (Fengler et al. 2015; Skeide et al. 2016; Skeide and Friederici 2016).

## Methods

This study was preregistered at https://osf.io/g9bke. The analyses follow the preregistration, and any additional analyses are explicitly marked as exploratory.

### Participants

MRI and behavioral data of 37 typically developing 3- and 4-year-old children (17 3-year-old children, mean age = 3.31, SD = 0.18, range = 3.07 to 3.59, 10 female; 20 4-year-old children, mean age = 4.31, SD = 0.17, range = 4.02 to 4.58, 11 female) were analyzed. Behavioral data from a standardized test battery of general language development (SETK 3–5; *Sprachentwicklungstest für drei- bis fünfjährige Kinder: SETK 3–5*; Grimm 2001) were available for a total of 60 monolingual German-speaking children, with 27 3-year-olds and 33 4-year-olds. Following our preregistration, we excluded children that had an indication for a speech development disorder (*n* = 2; *T*-value < 35 in the language test), and followed the exclusion criteria of Grosse Wiesmann et al. (2020) for the MRI analyses. That is, we excluded children that (i) did not participate in or aborted the MRI (*n* = 9), (ii) showed motion artifacts in the sMRI data, detected by visual inspection (*n* = 11) or (iii) had a coincidental neurological diagnosis (*n* = 1). In addition to these preregistered criteria, we had to exclude two more children whose language scores could not be computed as one child did not complete all subtests of the SETK 3–5 and the transcript of one subtest of another child had been lost. Degree of handedness was evaluated with the German version of the Edinburgh Handedness Inventory (Oldfield 1971). Parental informed consent was obtained for all children, and the study was approved by the Ethics Committee at the Faculty of Medicine of the University of Leipzig (number of approval: 090/12-ff). The data have previously been analyzed with regard to other research questions (Grosse Wiesmann et al. 2017a, 2017b, 2020; Berger et al. 2022).

### Behavioral data

#### Global language abilities

Children underwent a standardized test battery of general language development, designed for 3- to 5-year-old children (SETK 3–5; Grimm 2001). The SETK 3–5 is a comprehensive diagnostic tool that provides the possibility to compare individual test performance to detailed age-related norms for clinical application. In the current study, we were interested in relating individual developmental differences with brain structure. For this, we used raw values, rather than normalized *T*-scores, of each subtest of the SETK 3–5. To account for differences in the item structure of the test version for 3- versus 4-year-olds, we performed *z*-transformation within groups of 3- and 4-year-olds, respectively. The standardization was carried out within the age groups of the full sample (*n* = 60). The *z*-scores were combined to scales for language comprehension, production, and language memory, following the standard procedures in the SETK 3–5. In the last step, these scale values were aggregated to a global language score for each subject (see Table 1). Note, that, while these scores follow our preregistration, the names of the scores changed. A list of renaming of the scores compared to the preregistration can be found in the Supplementary Table S1. The global language scores of 3- and 4-year-olds were normally distributed, as indicated by a Shapiro–Wilk test (3-year-olds: *P* = 0.24, 4-year-olds: *P* = 0.98), and no outliers were identified.

**Table 1:** Mean and standard deviation of *z*-transformed global language and raw syntax scores of each age group.

	*N*	Mean	SD
Global language score—averaged *z*-values			
3 years	17	0.03	0.65
4 years	19	0.04	0.67
Sentence production score—raw values			
3 years	16	4.59	0.97
4 years	19	6.83	0.61
Sentence comprehension score—raw values			
3 years	17	14.29	2.93
4 years	20	11.35	1.79
Morpho-syntax score—raw values			
3 years	17	15.71	3.55
4 years	20	25.45	5.03

Note. To receive the global language scores, SETK 3–5 subtest scores were *z*-transformed and combined. Thus, only *z*-transformed global language scores are displayed. Raw syntax scores were *z*-transformed within each age group for further analyses.

#### Syntactic abilities

In order to assess complex sentence processing abilities, we developed and preregistered a coding procedure that quantified the syntactic complexity of produced sentences in two subtests of the SETK 3–5. This was achieved by identifying the length of the longest syntactically correct fragment in words per item, a proxy for syntactic production ability modified for children’s data (see Hunt 1970; Loban 1976; Nippold et al. 2014, 2017). Incomplete clauses with correct syntactic structure were included in the analysis (Vasilyeva et al. 2008). Hesitation and self-corrections were not considered as part of the fragment, but non-words with clear part-of-speech assignment and grammatically correct inflection were treated as real words. Two immediately following adjectives were counted as one to ensure that the length of the fragment would not be extended without adding syntactic complexity to it. The length of the fragments was computed based on production data collected in two standardized subtests of the SETK 3–5 that were designed to elicit syntactically complex sentences (see details in Supplementary Material; Grimm 2001). For the 3-year-olds, sentence production was elicited in a picture description task in which children were expected to produce prepositional phrases (SETK 3–5 subtest “Enkodierung semantischer Relationen”, ESR; Grimm 2001). For the 4-year-olds, sentence production was elicited in a sentence repetition task in which children were asked to reproduce sentences with correct morphosyntactic inflection and either plausible or implausible meaning (SETK 3–5 subtest “Satzgedächtnis”, SG; Grimm 2001). Importantly, our coding procedure was independent of the scoring system of the SETK 3–5 for these production subtests and was applied identically to both age groups. The raw scores (see Table 1) were *z*-transformed within the groups of 3- and 4-year-olds to account for differences in length of the syntactic fragment that may have arisen from differences in the elicitation context. This way, the length of 3-year-olds’ syntactically correct fragments was evaluated only in relation to the length of other 3-year-olds’ fragments, whereas 4-year-olds’ fragments length were evaluated in relation to those of other 4-year-olds. These standardized values formed the sentence production score. Testing sessions were audio recorded and language production data were transcribed. In addition, more than 20% of the audio files were transcribed by a second independent rater. Items that received conflicting transcriptions were transcribed by a third independent rater and the transcription of the majority was used. For the 3-year-olds, the first and second transcription agreed in over 87% and for the 4-year-olds in over 93% of the sentences. A Shapiro–Wilk test revealed that sentence production scores were normally distributed (3-year-olds: *P* = 0.50, 4-year-olds: *P* = 0.22) and had no statistical outliers. The sentence production score was used as our main preregistered measure for children’s sentence processing ability.

As additional exploratory indices of children’s syntactic ability, we had preregistered a sentence comprehension and a morpho-syntax score. To assess complex sentence comprehension, children were tested in an object manipulation task (SETK 3–5 subtest “Verstehen von Sätzen”; Grimm 2001). The presented sentences increase in their grammatical complexity (see details in Supplementary Material). We *z*-transformed raw scores (see Table 1) within age groups to form the sentence comprehension score. Sentence comprehension scores were normally distributed, as indicated by a Shapiro–Wilk test (3-year-olds: *P* = 0.069, 4-year-olds: *P* = 0.13), and no outliers were identified. The second exploratory measure estimated children’s knowledge of the morpho-syntactic rule system, which is fundamental to form grammatically correct sentences (Dittmar et al. 2008). As preregistered, this measure was obtained from the word generation subtest in which children were asked to assign the plural forms to nouns (SETK 3–5 subtest “Morphologische Regelbildung”; Grimm 2001). The resulting morpho-syntax scores (see Table 1) were normally distributed (3-year-olds: *P* = 0.068, 4-year-olds: *P* = 0.94) without statistical outliers.

#### General cognitive development

Children’s non-verbal IQ was assessed using the Kaufman Assessment Battery for Children (Kaufman ABC; Melchers and Preuß 2003), testing for visual and auditory working memory, selective attention as well as spatial representation abilities.

### MRI data acquisition

MRI data were obtained on a 3-T Siemens scanner (Siemens MRT Trio series) equipped with a 32-channel head coil. High-resolution 3D T_1_-weighted sMRI data were acquired with the MP2RAGE sequence (Marques et al. 2010) at 1.2 × 1 × 1 mm resolution with the following imaging parameters: inversion time TI_1_ = 700 ms; flip angle α_1_ = 4*^°^*; TI_2_ = 2,500 ms; α_2_ = 5*^°^*; repetition time TR = 5,000 ms; echo time TE = 3.24 ms; FoV = 192 *×* 192 mm; 176 sagittal slices; GRAPPA 3; partial Fourier phase factor 6/8; bandwidth 240 HzPx; acquisition time 5:22 min (as described in Grosse Wiesmann et al. 2020).

### MRI data analysis

#### Cortical surface-based analyses

We used the preprocessed brain images from recent studies using the current dataset to obtain measures of cortical thickness and surface area (see for details Grosse Wiesmann et al. 2020; Berger et al. 2022). In brief, individual brain images were preprocessed in FreeSurfer 5.3.0 (http://surfer.nmr.mgh.harvard.edu/) following the standard surface-based pipeline (Fischl and Dale 2000) to reconstruct cortical surfaces and generate local estimates of cortical thickness and surface area. Cortical thickness, defined as the closest distance from the gray matter/white matter boundary to the gray matter/cerebrospinal fluid boundary and surface area of the gray matter/white matter boundary was calculated at each vertex (Fischl and Dale 2000). The resulting maps for cortical thickness and surface area were smoothed on the tessellated surfaces using a 10-mm FWHM Gaussian kernel. A common group template was created from the individual T_1_-weighted images of the children who participated in the study using ANTs (Avants et al. 2008). To allow for an accurate matching of local cortical thickness and surface area measures across participants, the individual cortical surfaces were registered to the common group template.

#### ROI selection

Based on previous literature (Friederici and Gierhan 2013; Fedorenko and Thompson-Schill 2014; Qi et al. 2019) and following our preregistered hypotheses (https://osf.io/g9bke), we selected language-related ROIs in the fronto-temporal cortex of the left hemisphere, using the Desikan-Killiany atlas (Desikan et al. 2006). This atlas is implemented in the automated processing pipeline of FreeSurfer and its parcellation is based on neuroanatomical features of gyri (Desikan et al. 2006). The automated labeling shows high accuracy in comparison to manual parcellation. From the atlas labels, we created a language network mask, including the following areas (see Supplementary Fig. S1 and Supplementary Methods for a detailed description): superior temporal gyrus, banks of the superior temporal sulcus, middle temporal gyrus, transverse temporal cortex, temporal pole, inferior parietal lobule, supramarginal gyrus, insula, caudal middle frontal gyrus, and inferior frontal gyrus (pars opercularis, pars triangularis and pars orbitalis). In addition, we created a mask capturing the left BA44 that was our core hypothesized ROI for complex sentence processing (see Supplementary Fig. S1 and Supplementary Methods for a detailed description). For an exploratory analysis, we additionally created a mask of the left BA45 (see Supplementary Methods for a detailed description). All masks were generated on the common group template.

#### Statistical analysis

The relation of our main variables for global language and sentence production abilities with children’s cortical thickness and surface area were estimated in general linear models (GLM) using the tool mri_glmfit implemented in FreeSurfer. Our GLMs were designed to identify regions in which children’s global language or sentence production abilities were associated with the respective brain maturation marker (i.e. cortical thickness or surface area). Following our preregistration, we ran GLMs across both age groups of 3- and 4-year-olds with age group as factor and tested for an interaction between age group and children’s language scores. Significant interactions with age were followed-up with GLMs in the two age groups separately. In addition, for the purpose of comparison across the different measures, we also exploratorily tested the measures with non-significant age interaction in the two separate age groups. These analyses are marked as exploratory in the results section. To examine the specificity of the effects, we controlled for sex, non-verbal IQ, handedness, and estimated intracranial volume (eTIV). For all models, multiple comparison correction was applied with a clusterwise correction using the FreeSurfer tool mri_glmfit-sim, specifying a cluster-forming threshold of *P* < 0.01, clusterwise threshold of *P* < 0.05 (Greve and Fischl 2018), positive relation with surface area, and bidirectional relation with cortical thickness. For the clusterwise correction, a Monte Carlo simulation with 10,000 iterations was precomputed on the group template. In addition to clusterwise correction for multiple testing, *P* values were adjusted according to Bonferroni for the two brain maturation markers (i.e. cortical thickness and surface area). Moreover, in the models that included the additional syntax scores (i.e. sentence comprehension and morpho-syntax), we further Bonferroni-adjusted *P* values accordingly. We performed hypothesis-driven small-volume correction in pre-specified regions of interest. In particular, following the preregistration, we ran GLMs on children’s global language and sentence production scores in the language network mask. In addition, GLMs on the sentence production scores were run within the left BA44, following our a-priori hypotheses. Correlation coefficients for the relation between maturation indices and language scores were computed in RStudio (RStudio Team 2020). For this, each child’s mean cortical thickness/surface area from the significant clusters were extracted with FreeSurfer’s mris_anatomical_stats. These data were tested for normality using the Shapiro–Wilk test, which revealed some deviation from the normal distribution. Consequently, Spearman’s rank correlation coefficient was computed throughout the analyses. There were no statistical outliers with respect to mean thickness or surface area. In addition to our investigation of how language and sentence processing relate to cortical brain maturation in preschool children, we preregistered analyses on the relation with white matter connectivity using diffusion-weighted MRI data from the same children. The analyses of these data will be performed and reported separately.

## Results

To test whether language and sentence processing abilities were related to cortical brain structure, we reconstructed cortical surface area and thickness from high-resolution anatomical MRI in the same children that participated in the behavioral task battery. We ran GLMs to indicate cortical brain regions that show significant correlations with the global language and sentence production scores, respectively, including 3- and 4-year-old children in one model with age group as factor.

### Global language abilities and cortical brain structure

**Fig. 1:**
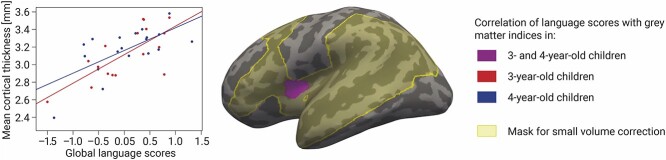
Linear correlation of global language abilities of 3- and 4-year-old children (violet) with cortical thickness in the preregistered mask of language-related ROIs (light yellow). The correlation was independent of age group, sex, non-verbal IQ, children’s laterality index for handedness and eTIV. The cluster is reported after multiple comparison correction at a cluster-forming threshold of *P* < 0.01 and clusterwise threshold of *P* < 0.05 and displayed on the inflated cortex of the common group template.

When performing small-volume correction in the language network mask, we observed a positive correlation of 3- and 4-year-old children’s global language scores with their cortical thickness in a cluster located in the anterior part of the insula extending to BA44 (see Fig. 1 and Table 2). This cluster remained significant when controlling for age group, sex, non-verbal IQ, handedness, and eTIV. There were no significant effects for surface area. Further, we observed a tentative interaction between age group and children’s global language scores in a cluster located in the posterior part of the left superior temporal gyrus (STG) bordering the Sylvian fissure (see Table 2). This effect was cluster-size corrected for multiple testing, but when additionally controlling for two brain measures (i.e. cortical thickness or surface area) according to Bonferroni, the interaction was only observed as a statistical trend. Following up on this trend, we ran separate models within each age group relating children’s global language scores with their cortical thickness and surface area. These analyses yielded no significant correlation in the individual age groups.

**Table 2:** Mask for SVC, anatomical region of effect, MNI coordinates, effect size, exact clusterwise *P* value, Bonferroni-corrected threshold for clusterwise *P* value, and cluster size of significant relations between cortical brain maturation indices and language scores in the left hemisphere.

	Mask for SVC	Anatomical region	Peak voxel coordinate in MNI 305 space (*X*, *Y*, *Z*)	Clusterwise *P* value	Corrected clusterwise threshold	Cluster-size (in mm^2^)
**Global language score**						
*Cortical thickness*						
3 years + 4 years	Language network	Insula, BA44^a^	−32.4, 4.4, 12.4	0.002	0.025	293.45
*Cortical thickness*						
3 years × 4 years	Language network	posterior STG	−58.5, −44.2, 26.7	0.042^*^	0.025	187.71
**Sentence production score**						
*Cortical thickness*						
3 years × 4 years	Language network	posterior STG	−65.3, −37.6, 12.0	0.030^*^	0.025	200.40
*Surface area*						
3 years	Language network	posterior STS^a^	−39.4, −61.6, 25.8	0.036^*^	0.025	376.54
*Cortical thickness*						
4 years	BA44	BA44	−35.3, 16.4, 21.7	0.009	0.025	50.09
**Sentence comprehension score**						
*Cortical thickness*						
4 years	BA44	BA44^a^	−35.3, 16.4, 21.7	<0.001	0.0125	108.08
**Morpho-syntax score**						
*Cortical thickness*						
4 years	BA44	BA44^a^	−38.3, 17.3, 21.4	0.003	0.0125	87.23

Note. All clusters are reported after multiple comparison correction at a cluster-forming threshold of *P* < 0.01 and clusterwise threshold of *P* < 0.05. It was controlled for sex, non-verbal IQ, and eTIV. Age group was included as factor in the models across both groups.

^a^These effects were independent of children’s laterality index for handedness.

^*^These *P* values are above the Bonferroni-corrected threshold for the clusterwise *P* value.

### Syntactic abilities and cortical brain structure

#### Sentence production score

**Fig. 2:**
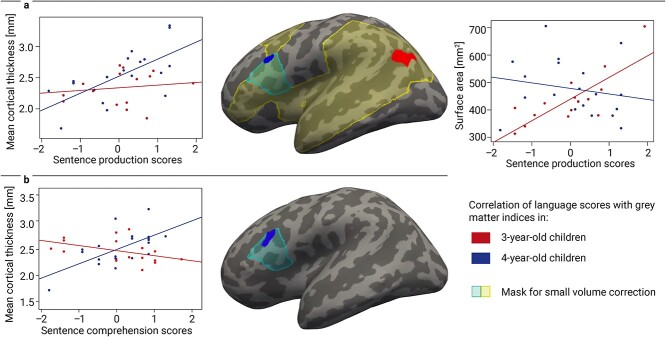
a) Linear correlation of sentence production abilities of children (3-year-olds: red; 4-year-olds: blue) with surface area/cortical thickness in the preregistered mask of language-related ROIs (light yellow) and of left BA44 (light blue). b) Linear correlation of sentence comprehension abilities of 4-year-old children (blue) with cortical thickness in the mask of left BA44 (light blue). The correlations were independent of sex, non-verbal IQ, and eTIV. All clusters are reported after multiple comparison correction at a cluster-forming threshold of *P* < 0.01 and clusterwise threshold of *P* < 0.05 and displayed on the inflated cortex of the common group template.

As main measure of sentence processing, we had preregistered the sentence production score. Our preregistered models showed no main effect with cortical thickness or surface area, neither in the language network mask nor in BA44. Within the language network mask, we found an interaction with age group in the left posterior STG (see Table 2), which only remained as a statistical trend after additional Bonferroni correction for two brain measures. Following up on this trend, we conducted separate analyses of 3- and 4-year-olds’ sentence production with their cortical brain structure. When performing small-volume correction in the language network mask, 3-year-olds’ but not 4-year-olds’ sentence production scores showed a tentative positive correlation with children’s surface area in the most posterior part of the superior temporal sulcus (STS; see Fig. 2a and Table 2). This effect was cluster-size corrected for multiple testing and remained significant when controlling for sex, non-verbal IQ, handedness, and eTIV, but was only observed as a statistical trend after additional Bonferroni correction for two brain measures. Further analyses corroborated that this relation was only present in the 3-year-olds (3-year-olds: ρ = 0.72, *P* = 0.002; 4-year-olds: ρ = −0.20, *P* = 0.40), and that the correlation coefficients in this region differed significantly between age groups (*z* = 4.67, *P* < 0.001). In the 4-year-olds, in turn, we observed a positive association of sentence production scores with cortical thickness with a small-volume correction in BA44 (see Fig. 2a and Table 2), and no effect for the 3-year-olds. Again, further analyses corroborated that this relation was only present in the 4-year-olds (3-year-olds: ρ = 0.17, *P* = 0.53; 4-year-olds: ρ = 0.69, *P* = 0.001), and that the correlation coefficients differed significantly between age groups (*z* = 2.79, *P* = 0.005). The cluster remained significant when controlling for sex, non-verbal IQ, and eTIV, but regressed after adding handedness to the model (see Supplementary Material). In an exploratory analysis reported in the Supplementary Material, we observed a positive correlation of 4-year-old but not 3-year-old children’s sentence production scores with their cortical thickness within a mask of BA45 (see Supplementary Fig. S2 and Supplementary Table S1).

#### Sentence comprehension score

As additional syntax measures, we had preregistered the sentence comprehension and morpho-syntax score, and further adjusted *P* values according to Bonferroni for these two scores in these analyses. We observed no significant cluster and no significant interaction effect for children’s sentence comprehension scores across both age groups with a small-volume correction in the language network mask or BA44. Exploratory, we ran separate GLMs for both age groups within the entire language network mask. This yielded no significant correlation of children’s sentence comprehension scores with their cortical thickness or surface area in neither of the two age groups. The exploratory GLMs in both age groups separately within the mask of BA44 yielded no significant effect for 3-year-olds’, but a positive correlation of 4-year-olds’ sentence comprehension scores with cortical thickness in BA44 (see Fig. 2b and Table 2). Further analyses confirmed that the positive correlation was only significant in the 4-year-olds (3-year-olds: ρ = −0.50, *P* = 0.039; 4-year-olds: ρ = 0.61, *P* = 0.004) and differed significantly between age groups (*z* = 5.31, *P* < 0.001). The effect remained significant when controlling for sex, non-verbal IQ, handedness, and eTIV.

#### Morpho-syntax score

We then tested for the relation of morpho-syntactic rule generation and cortical brain structure. No significant correlation or interaction of children’s morpho-syntax scores with their cortical thickness or surface area was found in our GLMs across both age groups in neither the language network mask nor BA44. When testing exploratorily in separate GLMs, we found no effect for the 3- or 4-year-olds within the language network mask. However, when testing exploratorily within BA44, we found a positive correlation between children’s morpho-syntax scores and their cortical thickness in the 4-year-olds, but no effect for the 3-year-olds (see Table 2). This effect remained significant when controlling for sex, non-verbal IQ, handedness and eTIV. Further analysis corroborated that the effect was only significant in the 4-year-old children (3-year-olds: ρ = −0.22, *P* = 0.40; 4-year-olds: ρ = 0.61, *P* = 0.004) and that the correlations differed significantly between age groups (*z* = 3.90, *P* < 0.001).

## Discussion

Between 3 and 4 years of age, important behavioral milestones in the acquisition of complex sentence structures are achieved (Fox and Grodzinsky 1998; Akhtar 1999; Tomasello and Brooks 1999; Guasti 2002; Kauschke 2012). So far, however, little was known about the cortical maturation underlying this important achievement in children’s language development. To address this, in the present study, we investigated the association of sentence processing abilities with cortical brain structure in 3- and 4-year-old children. Our results suggest that structural maturation of left BA44, a core region for syntax in adults, supports the emergence of complex sentence structures in 4-year-old children. Sentence processing abilities in 3-year-olds, in contrast, still rely on the maturation of a different brain region, namely the most posterior left STS involved in the integration of syntactic and semantic information in adults (Bornkessel et al. 2005; Friederici et al. 2009; Friederici 2011). The present findings suggest a qualitative shift in cortical structures relevant for sentence processing between 3 and 4 years, which may underlie children’s behavioral milestones in complex sentence processing around this age.

The early preschool years are a period of significant, potentially also qualitative, change in syntactic proficiency: By the age of 3, children can reliably process and produce the canonical word order of their language (Akhtar 1999; Dittmar et al. 2008; Schipke et al. 2012). Yet, they do not master more complex syntactic structures, such as passive constructions and subordinate clauses until 4 years of age (Fox and Grodzinsky 1998; Tomasello and Brooks 1999; Guasti 2002; Kauschke 2012). Conversely, a shift from item-based to abstract representation of syntactic constructions between 3 and 4 years has been proposed (Pinker et al. 1987; Akhtar 1999; Bidgood et al. 2020). In the current study, we show that this shift in syntactic development may be reflected in distinct relationships with different cortical areas in 3- compared to 4-year-olds. While we had hypothesized an association in left BA44 across both age groups, we found that complex sentence mastery was supported by this region only in 4-year-olds. This finding was consistent across all syntax measures, that is, in sentence production and comprehension as well as morpho-syntactic rule generation. In contrast, we did not find such a relation for the 3-year-olds. In the matured brain, left BA44 is known to support syntactic processes, such as hierarchical structure binding, and shows sensitivity to syntactic complexity, rule-generation and function words conveying structural cues (Ullman et al. 1997; Friederici et al. 2000, 2006; Friederici 2002, 2011; Tyler et al. 2005; Makuuchi et al. 2009; Newman et al. 2010; Goucha and Friederici 2015; Zaccarella et al. 2017). Between 4 and 9 years of age, functional activation of BA44 when processing sentences increases, but the neural activity is not yet modulated by syntactic complexity (Skeide et al. 2014, 2016). Our findings are in line with the suggestion that the functional prerequisites for the successful integration of syntax distinct from semantics may be present by 4 years of age, but not yet sufficient to master these demanding processing tasks separately (Skeide and Friederici 2016).

In contrast to our hypotheses, sentence processing proficiency in 3-year-old children was associated with structural maturation in a different region, namely, the most posterior left STS. In the adult brain, the posterior temporal cortex, consistently shows an activation in complex sentence processing together with the left IFG (Bornkessel et al. 2005; Friederici et al. 2009; Pallier et al. 2011; Goucha and Friederici 2015; Zaccarella et al. 2017) and has traditionally been associated with the integration of syntactic and semantic information (Bornkessel et al. 2005; Friederici et al. 2009; Friederici 2011). In the present study, the maturation of the most posterior STS was related to the longest syntactically correct fragment that 3-year-old children produced. From behavioral and neuroimaging studies, it is known that young children strongly rely on lexical meaning to successfully process complex sentence structures (Friederici 1983; Skeide et al. 2014; Strotseva-Feinschmidt et al. 2019). This behavioral finding is in line with an immature activation pattern with overlapping neural activity in the posterior superior temporal lobe for syntactic and semantic effects during sentence comprehension in 3- to 4-year-olds (Skeide et al. 2014). The observed association of the most posterior left STS may reflect this interaction between syntactic and semantic processes for producing longer and thus syntactically more complex utterances in 3-year-olds. For example, they may use specific sentence constructions based on the meaning of a known verb (Pinker et al. 1987; Akhtar 1999; Bidgood et al. 2020). Hence, structural maturation of the most posterior STS may contribute to young children’s sentence processing ability but alone may not be sufficient for the mastery of complex syntax. The present findings thus fit well with processing and production difficulties of 3-year-olds for more complex sentence structures (Fox and Grodzinsky 1998; Akhtar 1999; Tomasello and Brooks 1999; Guasti 2002; Kauschke 2012). The full mastery of complex syntax may depend on a larger network including the left IFG.

The mastery of the interaction of syntax and semantics in language development might also be reflected in the observed association between 4-year-olds’ sentence production and cortical maturation of BA45 (see Supplementary Results). This region functionally supports semantic processes in the adult brain (Friederici 2002; Goucha and Friederici 2015). Its structural involvement in children’s sentence production fits with the strong relationship between lexical knowledge and syntactic complexity in development (Marchman and Bates 1994; Moyle et al. 2007; Snedeker et al. 2012). A recent meta-analysis of developmental language comprehension studies reports, in addition to temporal activation, a shift from BA45 to BA44 between the age of 3 and 15 years (Enge et al. 2020). Extending previous imaging studies on the relation of left IFG maturation and complex sentence comprehension from 5 years (Fengler et al. 2015; Qi et al. 2019), we showed an association of BA44 already at 4 years with both sentence production and comprehension. This is consistent with a recent study that found broadly overlapping neural activity for sentence production and comprehension in adults (Giglio et al. 2021), and extends these findings to language acquisition. In contrast, this association was not present in 3-year-olds yet. The qualitative shift from the relevance of the most posterior STS in 3-year-olds to BA44 in 4-year-olds may facilitate the behavioral breakthrough observed in children’s syntax at around 4 years.

We further had hypothesized a relation between children’s global language abilities and cortical structure in the whole language network and observed a correlation with cortical thickness in the left anterior insula bordering BA44 within this network. In adults, the left insula is involved in a variety of cognitive processes including working memory demands, language comprehension and production (Flynn 1999; Mutschler et al. 2009; Kurth et al. 2010; Zaccarella and Friederici 2015). The association between structural changes in the insula and children’s global language performance across multiple linguistic domains fits with findings of its functional involvement in various language tasks in the mature brain. This finding suggests a gradual development of cortical structure relevant for more general aspects of language ability between 3 and 4 years of age, while a qualitative shift was observed for processing syntactically complex sentences.

To get a better understanding of the role semantics play in young preschoolers’ sentence production in particular, future research should investigate the relationship between children’s semantic production and the maturation of brain structure before the age of 4 years. This would require an experimental manipulation that reliably disentangles syntax from semantic abilities. An exciting question for future research is whether the qualitative shift in brain regions is related to performance or age. In functional MRI studies, subgroups of children showed differential activation pattern when subdivided by their performance (Nuñez et al. 2011; Knoll et al. 2012), which suggests that, in our studied age range as well, the observed shift may already occur earlier depending on individual proficiency. Moreover, it is still poorly understood how the left IFG and posterior temporal cortex as part of the language network develop and how this relates to language abilities including basic syntactic operations in children younger than 3 years. A limitation of the present study is that the results are correlational, and causal relations between sentence processing ability and brain development cannot be inferred. Future research may build on the present findings, for example, to conduct training studies to target causal effects. In addition, the current analyses and the delineation of cortical ROIs were defined based on a particular atlas (Desikan et al. 2006), which guided our definition of the brain structures. Future studies may evaluate how robust this definition is across different atlases.

## Conclusions

In sum, we showed that children’s sentence processing abilities were related to maturational changes in different brain regions in 3- compared to 4-year-olds, namely in the most posterior left STS for 3-year-olds but left BA44 for 4-year-olds. This suggests a qualitative shift toward more mature brain regions for complex sentence processing between 3 and 4 years of age, which may support syntactic milestones reached around 4 years.

## Supplementary Material

Supplementary_Material_final_bhac430

## Data Availability

All employed materials and data discussed in the paper are saved in a local repository at the Max Planck Institute for Human Cognitive and Brain Sciences, and data in fully anonymized format will be made available to the reader upon request (according to the data protection policy in the ethics agreement).
