# Supplementary material for: Children’s syntax is supported by the maturation of BA44 at 4 years, but of the posterior STS at 3 years of age
Source: Cereb Cortex. 2022 Nov 20;33(9):5426–35. doi: 10.1093/cercor/bhac430 (PMC10152089; doi:10.1093/cercor/bhac430)
Supplement: Supplementary_Material_final_bhac430 [file supplementary_material_final_bhac430.docx]

**Supplementary Material**

## **Children’s syntax is supported by the maturation of BA44 at 4 years, but of the posterior STS at 3 years of age**

## Running title: Syntax at 4 but not 3 years relates with BA44 maturation

Cheslie C. Klein^1^*^,^*^2*^, Philipp Berger^1^*^,^*^2^, Tomás Goucha^1^, Angela D. Friederici^1^ & Charlotte

Grosse Wiesmann^2^

^1^Department of Neuropsychology, Max Planck Institute for Human Cognitive and Brain

Sciences, Leipzig, Germany

^2^Research Group Milestones of Early Cognitive Development, Max Planck Institute for Human

Cognitive and Brain Sciences, Leipzig, Germany

***Correspondence to:**

Cheslie C. Klein

Max Planck Institute for Human Cognitive and Brain Science

Department of Neuropsychology

Stephanstraße 1a, 04103 Leipzig, Germany

Email: cklein@cbs.mpg.de

Phone number: +49 341 9940 157

Facsimile number: +49 341 9940 113

# Supplementary Table 1: Correspondence between reported and preregistered scores

**Table 1:** Name of language scores, original name as indicated in the preregistration and respective language measure retrieved from SETK 3-5 subtests.

| **Language score** | **Original name in preregistration** | **Language measure** |
| --- | --- | --- |
| Global language score | Global language development score | SETK 3-5 tasks covering language production, comprehension and memory – raw values of subtests standardized by age group and then averaged |
| Sentence production  score | Syntactic development score | Elicited production tasks – longest syntactically correct fragment in words standardized by age group |
| Sentence comprehension score | ‘Understanding sentences’ subtest score | Manipulation task – raw values standardized by age group |
| Morpho-syntax score | ‘Morphological rules’ subtest score | Morpho-syntactic rule generation task – raw values standardized by age group |

# Supplementary Methods: Description of SETK 3-5 tasks

The SETK 3-5 subtest ‘Encoding semantic relations’ (orig. ‘Enkodierung semantischer Relationen’) is a picture description task in which people and animals perform an action with spatial relation to an object. Therefore, the use of prepositional phrases is elicited in 3-year-olds with varying degrees of difficulty depending on the required preposition (Grimm 1975). In the subtest ‘Sentence memory’ (orig. ‘Satzgedächtnis’, SG), 4-year-olds are asked to reproduce sentences which consist of six to ten words with correct morphosyntactic inflection and either plausible (SG: “Lena lacht, nachdem sie gekitzelt wurde.” [engl. “Lena laughs after being tickled.”]; Grimm 2001) or implausible meaning (SG: “Ein frecher Fußball, der den alten Kasper heiratet, ist müde.” [engl. “A cheeky soccer ball marrying the old Punch is tired.”]; Grimm 2001). The length of the sentences is chosen, such that they cannot be retrieved solely from the working memory of the children but have to be reconstructed using the child’s grammatical and syntactic knowledge (Grimm 2001). This effect is further supported by sentences with implausible meaning, since the children cannot rely on their real-world knowledge (Grimm 2001). The subtest ‘Understanding sentences’ (orig. ‘Verstehen von Sätzen’, VS) consists of manipulation tasks: The children are instructed, for example, to move objects in a certain order or to touch one object with another. The grammatical complexity of the sentence structure increases from item to item, for instance by presenting the instruction in reverse order than the requested action (VS: “Gib mir die Kiste, nachdem du einen Knopf reingelegt hast.” [engl. “Give me the box after you put a button in it.”]; Grimm 2001), involving causal relations between two actions and including not only subject- but also object-initial sentences (VS: “Zeig mir: Der gelbe Ball, den der weiße Ball anstößt, fällt vom Tisch.” [engl. “Show me: The yellow ball bumped by the white ball falls off the table.”]; Grimm 2001). Some items overlap in the tests for 3- and 4-year-old children, but some of the more complex constructions occur only in 4-year-olds as, for example, object-first subordinate clauses are not understood by 3-year-olds (Grimm 2001; Schipke et al. 2012). Instead, the 3-year-olds additionally perform a sentence-picture-matching task in which they are asked to select one of four pictures that matches a sentence. In the subtest ‘Morphological rules’ (orig. ‘Morphologische Regelbildung’) children are asked to form the plural of real words. In addition, 4-year-olds are asked to build the plural of pseudowords. The test determines the acquisition level of the morphological rule system for plural formation (Grimm 2001).

# Supplementary Methods: Delineation of ROIs

We created a preregistered mask capturing the entire language network from the gyri-based Desikan-Killiany atlas implemented in the processing pipeline of FreeSurfer (Desikan et al. 2006). The area included in the language network mask contained the following gyri and sulci (see Desikan et al. 2006 for a full description): superior temporal gyrus with the Sylvian Fissure as upper boundary; middle temporal gyrus with the inferior temporal sulcus as lower boundary; inferior parietal lobule including the angular gyrus with the superior parietal gyrus as upper boundary; supramarginal gyrus with the intraparietal sulcus as upper boundary and the Sylvian fissure as lower boundary; inferior frontal gyrus with the inferior frontal sulcus as upper boundary, the orbital sulcus and circular insular sulcus as lower boundary and the precentral gyrus as posterior boundary; caudal middle frontal gyrus with the superior frontal sulcus as upper boundary, bordering anteriorly the middle frontal gyrus and posteriorly on the precentral gyrus.

In addition, we created a mask capturing the left BA44 which was parcellated as the gyrus anteriorly to the precentral gyrus, delineated dorsally by the inferior frontal sulcus and ventrally by the circular insula sulcus (Desikan et al. 2006). The mask of the left BA45, additionally created for the exploratory analysis, was labelled as the second gyrus anterior to the precentral gyrus (see Desikan et al. 2006 for a full description).

# Supplementary Figure 1: Masks for small volume correction

#
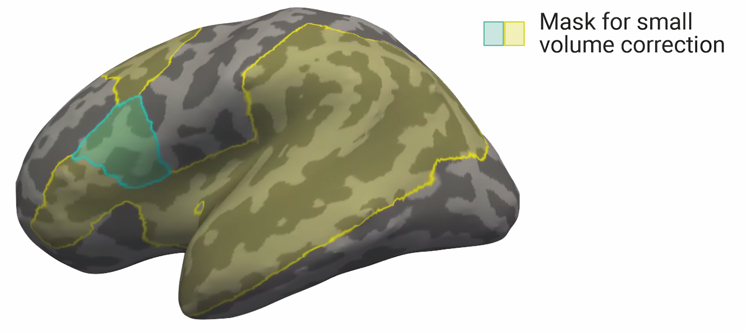


**Figure 1:** Mask of language-related ROIs in the frontotemporal cortex of the left hemisphere using the Desikan-Killiany atlas (light yellow; Desikan et al. 2006) and mask of BA44 for small volume correction (light blue).

**Supplementary Results: Preregistered analysis of the language memory scale in the language-related brain network**

We had also preregistered an additional analysis to investigate the relation between children’s cortical maturation indices (i.e., cortical thickness and surface area) and their overall performance in the SETK 3-5 subtests assigned to the language memory scale (Grimm 2001). For the 3-year-olds, this scale consists of the subtest ‘Phonological working memory for nonwords’ (orig. ‘Phonologisches Arbeitsgedächtnis für Nichtwörter’), in which children were asked to repeat nonwords of various length. For the 4-year-olds, the subtests ‘Memory span for word sequences’ (orig. ‘Gedächtnisspanne für Wortfolgen’), a repetition task with word lists, and ‘Sentence memory’ (orig. ‘Satzgedächtnis’) count in addition to the language memory scale. When performing small-volume correction in the language network mask, no significant main effect or interaction was found with cortical thickness and surface area in neither GLM across both age groups or 3- and 4-year-olds separately.

# Supplementary Results: Further analyses with right-handed children

Sentence production scores of the 4-year-old children were positively correlated with cortical thickness in the left BA44. The effect regressed when controlling for children’s laterality index for handedness. To follow up on the effect of handedness, we therefore tested only 4-year-olds with a clear indication for right-handedness (*n* = 14, mean = 71.5, SD = 16.88, range = 42.9 to 100, 7 female). This revealed a significant correlation of children’s sentence production scores with their cortical thickness in the same ROI. The follow-up analyses indicate that this effect was driven by right-handed children.

# Supplementary Results: Exploratory analysis of children’s sentence production abilities in BA45

In an additional analysis, we tested for the relation between children’s sentence production abilities and their cortical structures in the left BA45, as this area has been found to be functionally involved in sentence processing in preschool children and older (Skeide et al. 2014; Wu et al. 2016). No significant correlation of children’s sentence production scores with their cortical thickness or surface area, or interaction was found in our GLMs across both age groups in BA45. However, when testing 3- and 4-year-olds’ sentence production scores separately in this ROI, we found that 4-year-old, but not 3-year-old children showed a positive correlation with their cortical thickness in BA45 (see Supplementary Figure 2 and Supplementary Table 2). Further analysis corroborated that this relation was only present in the 4-year-olds (3-year-olds: *ρ* = -0.13, *P* = 0.62; 4-year-olds: *ρ* = 0.72, *P* < 0.001) and that the correlation differed significantly between age groups (*z* = 4.32, *P* < 0.001). This effect remained significant when controlling for sex, non-verbal IQ, handedness and eTIV.

# Supplementary Table 2: Exploratory analysis of children’s sentence production abilities in BA45

**Table 2:** Mask for small-volume correction (SVC), anatomical region of effect, MNI coordinates, effect size, exact clusterwise *P* value, Bonferroni-corrected threshold for clusterwise *P* value and cluster size of significant relation between cortical brain maturation index and language score in the left hemisphere.

|  | Mask for  SVC | Anatomical region | Peak voxel coordinate in MNI 305 space (X, Y, Z) | Clusterwise *P* value | Corrected clusterwise threshold | Cluster-size (in mm^2^) |  |
| --- | --- | --- | --- | --- | --- | --- | --- |
| **Sentence production score** | | |  |  |  |  |  |
| *Cortical thickness* | |  |  |  |  |  |  |
| 4y | BA44 | BA45 | -40.2, 36.9, -3.8 | 0.025 | 0.025 | 67.48 |  |

Note. Cluster is reported after multiple comparison correction at a cluster-forming threshold of *P* < 0.01 and clusterwise threshold of *P* < 0.05. It was controlled for sex, non-verbal IQ, handedness and eTIV.

# Supplementary Figure 2: Exploratory analysis of children’s sentence production abilities in BA45

#
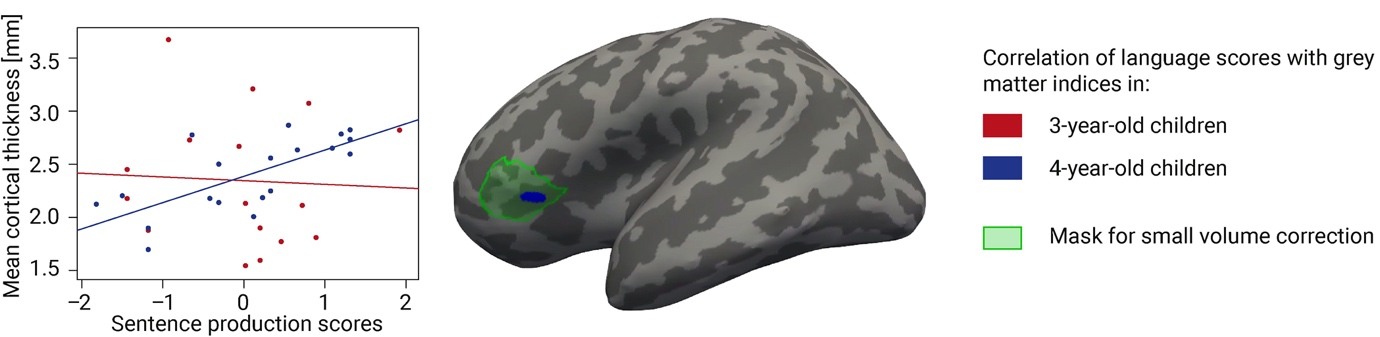


**Figure 2:** Linear correlation of sentence production abilities of 4-year-old children with cortical thickness in the mask of BA45 (light green). The correlation was independent of sex, non-verbal IQ, children’s laterality index for handedness and eTIV. The cluster is reported after multiple comparison correction at a cluster-forming threshold of *P* < 0.01 and clusterwise threshold of *P* < 0.05 and displayed on the inflated cortex of the common group template.

# Supplementary References

Desikan RS, Ségonne F, Fischl B, Quinn BT, Dickerson BC, Blacker D, Buckner RL, Dale AM, Maguire RP, Hyman BT, Albert MS, Killiany RJ. 2006. An automated labeling system for subdividing the human cerebral cortex on MRI scans into gyral based regions of interest. NeuroImage. 31:968–980.

Dittmar M, Abbot-Smith K, Lieven E, Tomasello M. 2008. German Childrens Comprehension of Word Order and Case Marking in Causative Sentences. Child Dev. 79:1152–1167.

Grimm H. 1975. On the Child’s Acquisition of Semantic Structure Underlying the Wordfield of Prepositions. Lang Speech. 18:97–119.

Grimm H. 2001. Sprachentwicklungstest für drei- bis fünfjährige Kinder: SETK 3–5. Diagnose von Sprachverarbeitungsfähigkeiten und auditiven Gedächtnisleistungen. Hogrefe: Verlag für Psychologie.

Schipke CS, Knoll LJ, Friederici AD, Oberecker R. 2012. Preschool children’s interpretation of object-initial sentences: Neural correlates of their behavioral performance: Children’s interpretation of object-initial sentences. Dev Sci. 15:762–774.

Skeide MA, Brauer J, Friederici AD. 2014. Syntax gradually segregates from semantics in the developing brain. NeuroImage. 100:106–111.

Wu C-Y, Vissiennon K, Friederici AD, Brauer J. 2016. Preschoolers’ brains rely on semantic cues prior to the mastery of syntax during sentence comprehension. NeuroImage. 126:256–266.
